# Inhibitory Effect of *Duabanga grandiflora* on MRSA Biofilm Formation via Prevention of Cell-Surface Attachment and PBP2a Production

**DOI:** 10.3390/molecules20034473

**Published:** 2015-03-10

**Authors:** Carolina Santiago, Kuan-Hon Lim, Hwei-San Loh, Kang Nee Ting

**Affiliations:** Faculty of Science, University of Nottingham Malaysia Campus, Jalan Broga, Semenyih, Selangor 43500, Malaysia; E-Mails: Carolina.Santiago@nottingham.edu.my (C.S.); kuanhon.lim@nottingham.edu.my (K.-H.L.); Sandy.Loh@nottingham.edu.my (H.-S.L.)

**Keywords:** MRSA, biofilms, *Duabanga grandiflora*, cell-surface attachment, PBP2a

## Abstract

Formation of biofilms is a major factor for nosocomial infections associated with methicillin-resistance *Staphylococcus aureus* (MRSA). This study was carried out to determine the ability of a fraction, F-10, derived from the plant *Duabanga grandiflora* to inhibit MRSA biofilm formation. Inhibition of biofilm production and microtiter attachment assays were employed to study the anti-biofilm activity of F-10, while latex agglutination test was performed to study the influence of F-10 on penicillin-binding protein 2a (PBP2a) level in MRSA biofilm. PBP2a is a protein that confers resistance to beta-lactam antibiotics. The results showed that, F-10 at minimum inhibitory concentration (MIC, 0.75 mg/mL) inhibited biofilm production by 66.10%; inhibited cell-surface attachment by more than 95%; and a reduced PBP2a level in the MRSA biofilm was observed. Although ampicilin was more effective in inhibiting biofilm production (MIC of 0.05 mg/mL, 84.49%) compared to F-10, the antibiotic was less effective in preventing cell-surface attachment. A higher level of PBP2a was detected in ampicillin-treated MRSA showing the development of further resistance in these colonies. This study has shown that F-10 possesses anti-biofilm activity, which can be attributed to its ability to reduce cell-surface attachment and attenuate the level of PBP2a that we postulated to play a crucial role in mediating biofilm formation.

## 1. Introduction

A number of reports have shown that bacterial cells growing in biofilms are profoundly resistant to many antibiotics. Biofilms play an intrinsic role in protecting bacterial cells from any fluctuations of the environment, including protecting the colonies from any potential antimicrobial agents [1]. It is well studied that the physiological properties of sessile biofilm populations are different from their planktonic counterparts and contribute to their better survival within the infected hosts. Biofilm- protected bacterial cells present a different mode of growth compared to planktonic cells, and the peculiarity of the mode of growth contributes to manifestation of antibiotic resistance. Due to this reason, treatment for biofilm-related infection becomes increasingly challenging, leading eventually to chronic device-related infections [2,3]. The biofilm forming ability of methicillin-resistance *Staphylococcus aureus* (MRSA) represents a major factor for nosocomial infections [1] and treatments for these infections are further complicated by the presence of other virulent factors such as toxin production and host immune evasion ability [4].

There are essentially two major steps in biofilm production: (1) cell-surface attachment, in which the bacteria attach to a surface in order to form colonies and this is also known as the primary attachment step; and (2) cell-cell interaction, which is an accumulative phase where the bacteria form microcolonies for construction of multilayer structure leading to biofilm development [5,6,7]. Biofilm formation in MRSA was previously reported to be mediated by PBP2a. PBP2a is an altered protein that evades antimicrobial action of beta-lactam antibiotics due to its low binding affinity [8]. It was hypothesized that PBP2a facilitates cell-cell interactions in the development of biofilm [9]. Hence, development of anti-biofilm agents that interfere with steps involved in biofilm formation and disrupt PBP2a expression would be a sensible approach in developing a new treatment for recalcitrant MRSA infections.

*Duabanga grandiflora* has been used traditionally to treat patients with skin conditions such as eczema or atopic dermatitis, who are also generally predisposed to *Staphylococcus aureus* infections [10,11]. In our earlier studies, we have reported that the ethyl acetate extract of the leaves of *D. grandiflora* possessed a broad spectrum antimicrobial action against a number of bacterial strains including MRSA [12,13]. The current study is aimed to investigate the effects of F-10, a bioactive fraction isolated from the leaves of *D. grandiflora*, on MRSA biofilm forming capacity and PBP2a expression in the biofilm.

## 2. Results and Discussion

### 2.1. Results

**Figure 1:**
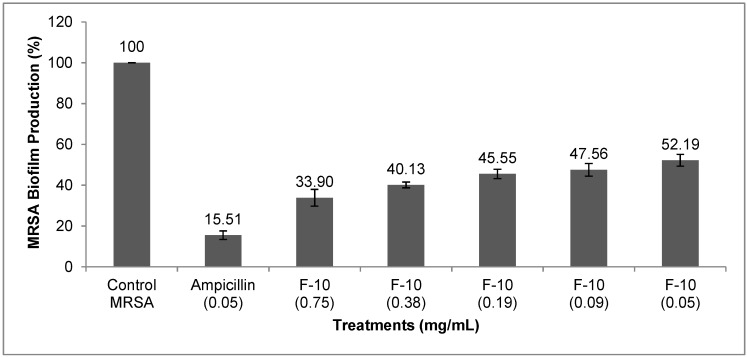
MRSA biofilm formation (%) in microtiter plate wells containing F-10 (mg/mL) treatments. Three wells were used for each treatment. Experiment is representative of three independent tests, and error bars indicate the standard deviation. All difference between control and treated MRSA were statistically significant (*p* < 0.001).

F-10 was tested at concentrations ranging from 0.75 mg/mL to 0.05 mg/mL and ampicillin at 0.05 mg/mL. The concentrations, 0.75 mg/mL and 0.05 mg/mL, were the minimum inhibitory concentrations (MICs) against MRSA growth in planktonic state for F-10 and ampicillin, respectively [14]. Figure 1 shows percentage of MRSA biofilm formation in the different treatments. Overall, F-10 exhibited appreciable activity against MRSA biofilm production, whereby biofilm production was at least 2-fold lower compared to control MRSA, while at the highest tested concentration of F-10 (0.75 mg/mL), the production of biofilm was down to 33.90%. On the other hand, ampicillin (0.05 mg/mL) reduced the biofilm production by more than 80% (Figure 1).

**Figure 2:**
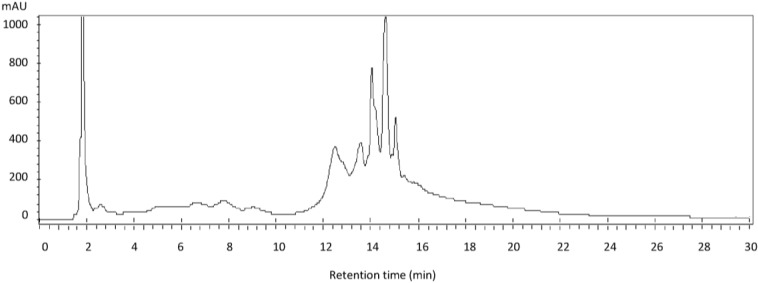
HPLC chromatogram of F-10 (40 µL of 10 mg/mL, C_18_-reversed phase, 4.6 × 150 mm, 5 μm, detected at 254 nm) showing the presence of multiple severely overlapped peaks.

Phytochemical analysis revealed the presence of alkaloids, tannins, saponins, steroids, glycosides and flavonoids in F-10. Preliminary HPLC analysis on the other hand confirmed that F-10 comprised a complex mixture of compounds (Figure 2).

**Figure 3:**
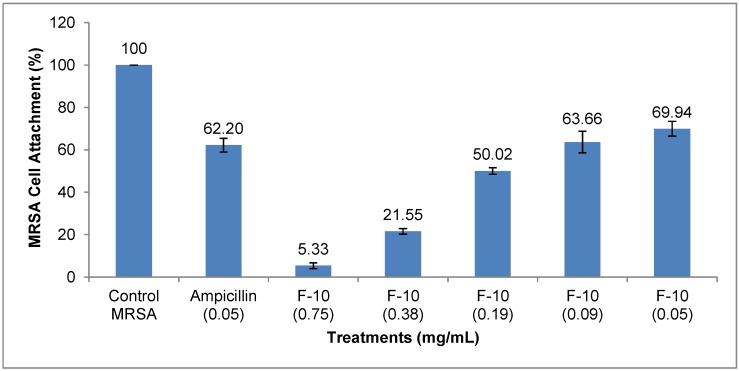
Attachment of MRSA cells to the surfaces of microtiter plate wells containing F-10 and ampicillin. Three wells were used for each treatment. Experiment is representative of three independent tests, and error bars indicate the standard deviation. All differences between control and treated MRSA were statistically significant (*p* < 0.001).

In order to shed some light on the mechanism of anti-biofilm activity of F-10, cell-surface attachment was studied where MRSA cultures, treated either with ampicillin or F-10, were incubated for an hour. Cultures treated with F-10 showed a concentration dependent reduction in cell-surface attachment. Notably, at MIC of F-10 cell-surface attachment was markedly prevented (*i.e*., 5.33%), but the same cannot be said for that of ampicillin at MIC, which showed 62.20% of cell-surface attachment (Figure 3).

**Figure 4:**
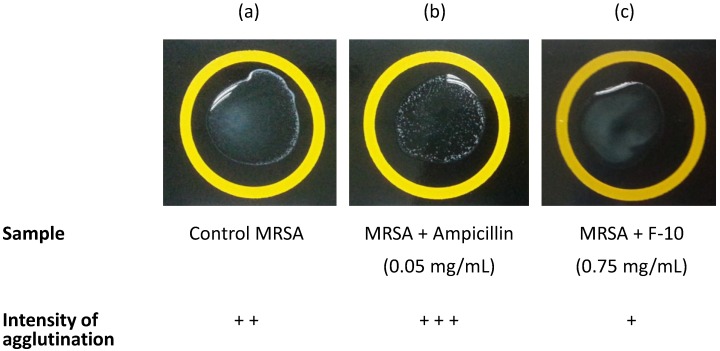
Representative images of PBP2a agglutination on MRSA biofilms showing (**a**) untreated control MRSA; (**b**) MRSA treated with ampicillin (0.05 mg/mL) and (**c**) MRSA treated with F-10 (0.75 mg/mL). Image (b) showed the highest intensity of agglutination followed by (a) and (c). (Interpretation: + + + high, + + moderate, + low).

A PBP2a latex agglutination test was employed to measure semi-quantitatively the level of PBP2a found in the biofilm. A higher intensity of agglutination observed essentially corresponds to a higher level of PBP2a found in the biofilm. MRSA control showed a moderate intensity of agglutination while ampicillin treatment appeared to increase the amount of PBB2a in the biofilm. However, treatment with F-10, in all the concentrations used, resulted in a much lower agglutination of PBP2a (Table 1 and Figure 4).

**Table 1:** Semi-quantitative estimation of PBP2a occurrence in biofilms isolated from different treatments. Intensity of agglutination against a turbid background was observed and scored between + to + + +, where the control latex which showed no reactivity in the absence of PBP2a is considered as “−” (interpretation: + + + high, + + moderate, + low, *n* = 3).

Treatments (mg/mL)	Intensity of PBP2a Agglutination
Control MRSA	+ +
Ampicillin (0.05)	+ + +
F-10 (0.75)	+
F-10 (0.38)	+
F-10 (0.19)	+
F-10 (0.09)	+
F-10 (0.05)	+

### 2.2. Discussion

Our study revealed that F-10, a semi-purified fraction isolated from *D. grandiflora*, possessed promising anti-MRSA activity via inhibition of biofilm production. From our preliminary phytochemical analysis of this fraction, the major classes of phytochemicals detected, which include alkaloids, tannins, saponins, steroids, glycosides and flavonoids, are known to be associated with antibacterial effects [15]. For instance, flavonoids are known to affect the sortase activity, which is known to influence the adhesive property of bacterial cell wall, leading to interruption of biofilm development [16,17]. Therefore, the occurrence of flavonoids in F-10 could represent a plausible explanation for the observed reduction of MRSA biofilm production when the cultures were treated with F-10. The anti-biofilm property of F-10 was further supported by the microtiter attachment assay results which showed that cell-surface attachment was effectively inhibited by F-10 (substantially better than ampicillin). This is a noteworthy finding, since the percentage of bacterial cell-surface attachment is directly proportional to the final mass of biofilm formed. A lower percentage of cell-surface attachment therefore corresponds to a lesser number of bacterial cells involved in biofilm development, which essentially gives rise to formation of weaker biofilm structures [18], and making the bacteria more vulnerable to eradication. Although ampicillin was found to suppress MRSA biofilm production, its inability to prevent cell-surface attachment within the first hour suggested a delayed antibacterial action. Previous studies have reported that a delayed antibacterial action may lead to prolonged and repeated exposure of the antibiotic to the bacteria, which promotes emergence of multidrug resistance [19,20]. Furthermore, the MIC of ampicillin used to treat the MRSA cultures was 50 μg/mL which exceeded the MIC breakpoints for susceptibility to beta-lactam drugs [21]. Therefore, ampicillin is not indicated for treatment of biofilm related infections.

In the PBP2a latex agglutination test, F-10 was observed to reduce the level of the resistant protein, PBP2a, in the MRSA biofilm. In a separate study, we have shown that F-10 attenuated the level of PBP2a in MRSA from a Western blot experiment [14]. It was previously suggested that the expression of high levels of PBP2a in MRSA could potentially promote cell-cell interactions that are not occurring between methicillin-sensitive *Staphylococcus aureus* (MSSA) cells, although the mechanism by which PBP2a promotes biofilm production remains unknown. It is well known that cell-cell interactions are vital for multilayer structure formation in development of biofilm [9,19]. We therefore postulated that the attenuation of PBP2a levels in MRSA by F-10, which will then negatively affect cell-cell interaction between MRSA cells that are involved in development of multilayer structures, consecutively contributed to the reduction in biofilm production.

## 3. Experimental Section

### 3.1. Plant Material

*D. grandiflora* leaves were collected from a growing tree in Simpang Pulai, Pahang, Malaysia (GPS location: N04° 33.701' E101° 11.685'). Herbarium voucher specimens (herbarium code UNMC75) are deposited at the Herbarium of Faculty of Science, University of Nottingham Malaysia Campus.

### 3.2. Extraction and Isolation

The ground leaf material was subjected to sequential extraction using hexane (He), ethyl acetate (EA), and ethanol (EtOH), as described elsewhere [13]. The ethyl acetate extract was fractionated by using a combination of vacuum liquid chromatography and preparative centrifugal thin layer chromatography methods (silica gel). The solvent system used for elution was CHCl_3_ in decreasing amount of hexane or CHCl_3_ in increasing amount of MeOH, *i.e.*, He/CHCl_3_ (1:1) → CHCl_3_ (100%) → CHCl_3_/MeOH (3%) → CHCl_3_/MeOH(5%) → CHCl_3_/MeOH (7%) → CHCl_3_/MeOH (10%) → CHCl_3_/MeOH (15%). The column was finally flushed with EtOH. Fraction F-10 was eluted when the solvent system CHCl_3_/MeOH (15%) was used.

### 3.3. Phytochemical and HPLC Analysis

Phytochemical analysis of F-10 was carried out according to methods described previously [22]. An aliquot of F-10 (40 μL of 10 mg/mL) was analyzed by C_18_-reversed phase HPLC using the following gradient solvent system: 2 min at 10% acetonitrile (ACN)/miliQ water (H_2_O); a linear gradient to 75% ACN/H_2_O over 12 min; isocratic at 75% for 10 min; a linear gradient to 100% ACN for 2 min; isocratic at 100% ACN for 4 min. HPLC was performed on a Varian 940-LC system using a reversed phase analytical column (Pursuit XRs C_18_, 4.6 × 150 mm, 5 µm) with photodiode array (PDA) detection at 254 nm.

### 3.4. Bacterial Strain, Growth Conditions and Determination of MIC Ampicillin

The bacterial strain used in this study was MRSA ATCC 43300. The strain was maintained on tryptic soy agar (TSA) (Hi-Media, Mumbai, India) supplemented with 2% NaCl (Merck, Darmstadt, Germany). All the experiments were initiated using fresh overnight cultures grown in tryptic soy broth (TSB) (Hi-Media) containing 1% glucose (Merck). MICs of ampicillin and F-10 against MRSA is 0.05 mg/mL and 0.75 mg/mL, respectively, which were determined based on methods described previously [14,23].

### 3.5. Inhibition of Biofilm Assay

Experiments were conducted based on methods described previously [20]. Basically, a 96-well microtiter plate was prepared with F-10 at the following concentrations; 0.75 mg/mL, 0.38 mg/mL, 0.19 mg/mL, 0.09 mg/mL, and 0.05 mg/mL, and ampicillin at 0.05 mg/mL. Aliquots of MRSA suspension were added to these wells. Final inoculum size was 1 × 10^5^ CFU/mL in total volume of 200 μL in each well. The plate was incubated for 24 h at 35 °C. After incubation, the wells were washed with physiological buffered saline (PBS) solution and quantification of biofilm production was established by crystal violet staining method [24]. Experiment was done in triplicates on three separate occasions.

### 3.6. Microtiter Attachment Assay

Experiment were conducted based on methods previously described by Overhage *et al.* [18]. A 96-well microtiter plate was prepared with F-10 at the following concentrations; 0.75 mg/mL, 0.38 mg/mL, 0.19 mg/mL, 0.09 mg/mL, and 0.05 mg/mL, and ampicillin at 0.05 mg/mL. Aliquots of MRSA suspension were added to these wells. Final inoculum size was 1 × 10^7^ CFU/mL. The plate was incubated for 1 h at 35 °C. Following incubation, the wells were washed with PBS and percentage of cell attachment was determined by crystal violet staining method [24]. Experiment was done in triplicates on three separate occasions.

### 3.7. PBP2a Latex Agglutination Test on MRSA Biofilm

MRSA was cultured in 50 mm diameter Petri dishes in 10 mL of TSB +1% glucose supplemented with F-10 at concentrations ranging from 0.05–0.75 mg/mL and ampicillin at 0.05 mg/mL. The petri dishes were incubated for 24 h at 35 °C. After incubation, the broth was carefully removed and 0.5 mL PBS was added to the petri dishes. Using a sterile 5 µL inoculating loop, the biofilm layer was scraped off just to fill the internal diameter (gives approximately 1.5 × 10^9^ CFU/mL). The obtained bacterial biofilm was processed according to manufacturer’s instructions of MRSA screening kit (Cat. No. DR900A Denka Seiken, Tokyo, Japan) in order to detect for the presence of PBP2a. Semi-quantitative estimation of PBP2a production in biofilms was done based on protocols described by Zhao *et al.* [25], in which the intensity of agglutination was observed and scored between + and + + +, where the control latex which showed no reactivity in the absence of PBP2a was scored as −.

### 3.8. Statistical Analysis

Results for inhibition of biofilm and microtiter attachment assays were shown as means ± standard deviation of three independent experiments. A one-way analysis of variance with Bonferroni multiple comparison tests were used to compare for difference between the control and treated groups. A *p* value of 0.001 was taken as statistically significant [20].

## 4. Conclusions

We conclude that F-10, the bioactive fraction obtained from *D. grandiflora*, inhibited biofilm formation in MRSA by preventing cell-surface attachment and interrupting PBP2a expression, which indirectly disrupts cell-cell interaction that is also necessary for biofilm development. Therefore it is postulated that PBP2a plays a crucial role in mediating the formation of biofilm which renders further resistance in these sessile biofilm populations of MRSA. Finally, some phytochemicals present in the bioactive fraction obtained from *D. grandiflora*, F-10, may be good candidates for development of new treatment for MRSA.
